# Prevalence and Intensity of Intestinal Helminth Infections in Preschool Pupils in Lugari Subcounty, Kakamega County, Kenya

**DOI:** 10.1155/2020/8871042

**Published:** 2020-11-07

**Authors:** Daniel Kevin Werunga, Elizabeth Nanjala Omukunda, Jackson Cheruiyot Korir

**Affiliations:** Department of Biological Sciences, Masinde Muliro University of Science and Technology, P.O. Box 190, Kakamega, Kenya

## Abstract

Intestinal helminths cause anaemia, malnutrition, indigestion disorders, retarded growth, and low mental abilities in pupils. About 1.5 billion are infected globally. Intestinal helminth infections are caused by *Ascaris lumbricoides*, *Trichuris trichiura*, *Strongyloides stercoralis*, *Enterobius vermicularis*, *Ancylostoma duodenale*, and *Necator americanus*. Lugari Subcounty has poor sanitation and inadequate clean water. This study determined the prevalence of intestinal helminth infections in preschool pupils in Lugari Subcounty. A stratified multistage cluster experimental design was used. Sampling was carried out in four wards: Lumakanda, Lugari, Luandeti, and Chekalini. Preschool pupils of either gender were selected randomly. Written consents and verbal assent were obtained from parents or guardians and preschool pupils, respectively. Questionnaires were administered in order to collect sociodemographic data. Stool samples were collected and tested for the presence of eggs using the standard Kato-Katz technique. Prevalence rate and prevalence ratio were calculated as the percentage of infected preschool pupils among the total number of preschool pupils examined. Preschool pupils positive with helminths were treated freely, and a follow-up screening was conducted three months after treatment. Approval of the study was sought from the Masinde Muliro University of Science and Technology Institutional Ethical Review Board (MMUST IRB). The overall prevalence of intestinal helminths was 12.3%. Only one species, *Ascaris lumbricoides*, was identified. Statistical tests were carried out at a 5% significance level (*p* < 0.05, confidence interval (CI) 95%). There was a statistically significant association for prevalence and intensity of intestinal helminths versus factors like school location, knowledge of washing hands before eating, and awareness of washing hands after visiting a toilet. Although this study revealed a low prevalence and light intensity, some factors had significant effects on intestinal helminth infections among the preschool children. Therefore, there is a need to intensify efforts for their intestinal helminth control.

## 1. Introduction

Over 270 million school pupils are located in regions with intensive intestinal helminthiases transmission, hence the need for protection, intervention, and treatment [1]. In Sub-Saharan Africa, it is estimated that about 1.98 billion individuals, including 40–50 million school-age pupils, are infected with intestinal helminths which results in a loss of about 7.5 million DALYs in the region against a global estimate of 22.1 million DALYs [2]. Primary pupils have the highest prevalence and infection intensities, and preschool pupils are likely to have as high infection rates of helminthiases as the primary pupils [3]. Kenya has more than 1.2 million pupils infected with intestinal helminths with 5 million at risk of infection. These infections are a salient public health problem among school pupils [4]. Intestinal helminthiases are common in developing countries and are of major health hazard because of their effect on the growth, development, nutritional, health, and immune status of the population [5–7]. Impacts on growth and development are common among pupils with heavier infections, whereas lighter infections may lead to stunted growth if the level of nutrition of the society is low [7] as a result of poor or nonabsorption of iron, potassium, and sodium ions in the body [8]. Intestinal helminths have been known to be the salient contributing factor of intestinal blood loss leading to iron deficiency and protein malnutrition [8]. Intestinal helminthiases are prevalent in most parts of Kenya with a variation in species distribution; for example, *N. americanus* is more prevalent at the coastal region of East Africa [9, 10]. In Kenya, intestinal helminthiases are widely distributed with prevalence ranging from 12 to above 90% and have been linked to anaemia [11–13].

Several studies done in Busia district revealed intestinal helminth prevalences of 90% and above among school pupils with *A. lumbricoides* and *T. trichiura* being common [14, 15], thus the need to adopt the school-based approach for management and treatment of helminthiasis. In Bondo and Kisumu districts, helminthiasis prevalences of 12.3% and 12.9%, respectively, [16, 17] were recorded. Kakamega County on the other hand recorded a higher prevalence of 43.5% [18]. Lugari Subcounty in Kakamega County in the Republic of Kenya has a large population of farmers who obtain domestic water from either rivers or wells. Shallow wells are the main source of water in the region. Some of these wells are covered while others are exposed, hence a risk for helminthiases. Majority of the preschool pupils come from low- to middle-income families and are likely to be exposed to intestinal helminthiases. Deworming programme was initiated as one of the control measures [19]. This study determined the prevalence and factors for intestinal helminth infections in preschool pupils in Lugari Subcounty.

## 2. Materials and Methods

### 2.1. Study Site

The study was carried out in Lugari Subcounty, a second-order administrative division located in Western Kenya 34°28′ to 35°00′ east and 0°25′ to 1°0′ north with an altitude of 1844 metres above sea level. It has a population of 215,920 and an area of 670 km^2^. Stratified multistage cluster design was used resulting into all the four wards of Lugari Subcounty: Lumakanda, Lugari, Luandeti, and Chekalini. All the four wards were picked for full representation. Preschool pupils were clustered according to sex, schools, wards, and environmental setup. Eight schools were chosen using the lottery method as suggested by the WHO guidelines [12]. Four schools were from the periurban setup and four from the rural setup. The sample size was determined according to Fox et al. [20]. The confidence level is 95% and precision of 5%. The prevalence previously determined in Busia of 91.7% [14, 20] was used to determine a sample size of 118 preschool pupils. However, 240 were recruited to take care of those who would likely drop out or fail to give a stool sample. There was a variation in the number of preschool pupils in different schools; thus, it was not possible to have a uniform number from each school. Therefore, all preschool pupils whose parents or guardians were willing to participate in the study were recruited.

### 2.2. Data Collection

Ethical approval was obtained from the Masinde Muliro University of Science and Technology Ethics Review Committee (MMU/COR: 403009(42)) and a research permit from the National Commission of Science, Technology and Innovation prior to the study (NACOSTI/P/16/92479/13634). Permission was sought from the county education and health offices. Finally, parents/guardians filled the informed consent forms before the commencement of the study. Pupils assented orally before the stool sample collection. Each participant and questionnaire was allocated a unique code for identification. Some of the questions asked including accessibility to latrine or toilet at home, a habit of hand washing after visiting latrine or toilet, were tackled by the respondents. Stool polypots were labelled with the unique code representing each of the participants. Stool samples were collected on a filter paper and transferred to labelled containers with the help of research assistants. The freshly collected stool samples were transported to the laboratory, approximately 50 kilometers (40 minutes of travelling) from the collection point, and examined on the same day of collection. Out of the original 204 participants who filled the questionnaire, 74 were not able to provide stool samples.

Only eggs were observed. Stool samples were processed according to the standard Kato-Katz method [21] in the laboratory by laboratory technologists and quality assurance by a senior laboratory technician, and results were recorded in a data sheet. The outcome of the stool was communicated to parents/guardians, and the provision of Albendazole by the study physician was made to pupils who tested positive.

### 2.3. Statistical Analysis

The coded data on the presence or absence of intestinal helminths in stool samples, number of eggs observed, and questionnaire was entered into the Microsoft Excel programme 2007 version and later imported into statistical software, STATISTICA 12. Prevalence ratio was used to calculate the strength of risk factors on infections of *Ascaris lumbricoides*. Prevalence ratios were calculated to relate the chances of infections with intestinal helminth according to Thompson, et al. [22]. Chi-square was run to determine the significant differences within variables, respectively [22]. Statistical tests were carried out at a 5% significance level (*p* < 0.05, confidence interval (CI) 95%). *p* < 0.05 was regarded as statistically significant.

## 3. Results

### 3.1. Prevalence of Helminthic Parasites

Out of 218 selected respondents, 93.6% (204) participated in giving information on the assessment of risk factors and only 63.7%(130) provided stool samples. Only *Ascaris lumbricoides* was present with a prevalence of 12.3% (Table 1). The results revealed that over fifty-five percent of the pupils, whose parents used water from uncovered wells and the river, were boys. Out of the 130 stool specimens obtained, 72.3% were from rural schools (Majengo, Sirende, Mapengo, and Chetambe) whereas 27.7% were from periurban schools (Lumakanda, Lugari, Musembe, and Maturu). Out of the 94 pupils in rural schools, 46.8% were boys and 53.2% were girls. Both boys and girls from the rural schools had an overall prevalence of 17% of *A. lumbricoides*. Girls had a prevalence of 14% of *A. lumbricoides* while boys had a prevalence of 20.5% of *A. lumbricoides*. The association between the prevalence of intestinal helminths and school location was statistically significant (*p* = 0.002). Out of the 36 pupils in periurban schools, 63.9% were boys and 36.1% were girls. None of the boys or girls from the periurban schools was infected with *A. lumbricoides* as indicated in Table 1.

### 3.2. Risk Factors for the Prevalence of *Ascaris lumbricoides*

No intestinal helminth infections were registered in periurban schools. This may be because pupils from periurban schools are more exposed to information about helminths than their counterparts from rural schools. Prevalence ratios were used to establish the magnitude of various risk factors for intestinal helminth infections. The whole subcounty had a prevalence of 12.3% of *A. lumbricoides* in both boys and girls. Pupils who were not aware that they are supposed to wash hands before eating are 5 times more likely to be infected with intestinal helminths than pupils who knew. The pupils who did not have any type of toilet at home were 4 times more likely to be infected with intestinal helminths than pupils who have toilets at home. The low prevalence may be due to the fact that only 2% of the respondents did not have knowledge on the source of infection of intestinal helminths. About twenty percent of the respondents did not deworm their children. Pupils without knowledge of washing hands after visiting the toilet were 4 times more likely to be infected with intestinal helminths than pupils who were aware. Pupils who used water from the river, more than one source, springs, and pipes are 4, 3, 2, and 2 times more likely to be infected with intestinal helminths than those who used water from both covered wells and uncovered wells. The number of pupils with intestinal helminths was 3-fold greater if a pupil used water that is not treated than those who used treated water.

**Figure 1:**
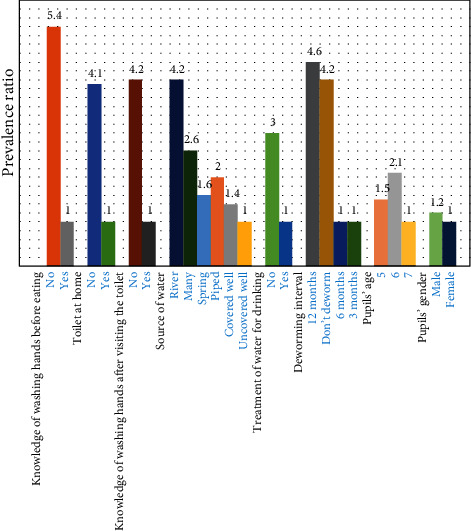
Association between prevalence and social factors that affect helminth infections.

Pupils who were dewormed at intervals of 3 and 6 months had equal chances of being infected with intestinal helminths. The number of intestinal helminth infections among pupils who were dewormed annually and those who were not dewormed was 5-fold and 4-fold greater than that of pupils who were dewormed at intervals of between 3 and 6 months (Figure 1).

Pupils who were 5 years old are 50% more likely to be infected with intestinal helminths than those who were 7 years old. Pupils who were 6 years old were 60% and were twice more likely to be infected with intestinal helminths than their colleagues who were 5 years and 7 years old, respectively. The number of boys with intestinal helminths is 20% greater than girls (Table 2).

Graphical representation of the association between the prevalence of *A. lumbricoides* and socioeconomic factors is shown in Figure 1.

Using the Chi-square test, a statistically significant association was established between the prevalence of intestinal helminths and school location (*X*^2^(1, *N* = 130) = 9.992, *p* = 0.002). The association was statistically significant between the prevalence of intestinal helminths and knowledge of washing hands before eating (*X*^2^(1, *N* = 130) = 15.331, *p* = 0.001). A statistically significant association was established between the prevalence of intestinal helminths and awareness of washing hands after visiting the toilet (*X*^2^(1, *N* = 130) = 10.382, *p* = 0.001). The following risk factors did not have a statistically significant association with the prevalence of intestinal helminths: method of faecal disposal, place of residence, source of water, water treatment method, deworming interval, pupils' age, and pupils' gender ((*X*^2^(3, *N* = 130) = 7.774, *p* = 0.051), (*X*^2^(1, *N* = 130) = 0.883, *p* = 0.347), (*X*^2^(5, *N* = 130) = 5.705, *p* = 0.336), (*X*^2^(4, *N* = 130) = 6.343, *p* = 0.175), (*X*^2^(4, *N* = 130) = 9.454, *p* = 0.051), (*X*^2^(4, *N* = 130) = 4.407, *p* = 0.354), and (*X*^2^(1, *N* = 130) = 0.162, *p* = 0.687), respectively (Table 3)).

### 3.3. Intensity of Helminthic Parasites

The intensity of infection for intestinal helminths was calculated by multiplying the number of observed eggs of *A. lumbricoides* by 24 to give eggs per gram of faeces (EPG) based on the WHO [23] grouping system of intestinal helminth infection intensities [24]. The intensity of infection was expressed as the mean eggs per gram of faeces (EPG). There were no stool samples from preschool pupils with more than 50000 EPG of *A. lumbricoides*, and therefore, heavy intensity was not encountered in the study site.

Out of the 16 study participants who tested positive with *Ascaris lumbricoides* at the subcounty level, two participants who had egg counts of 5000-49999 EPG were grouped under moderate intensity, and fourteen samples with 1-4999 EPG were grouped under light intensity as shown in Table 4.

Both boys and girls had an average egg count of 4498 EPG (light intensity). Out of the 9 infected boys, 8 had light intensity and only 1 had moderate intensity. All the boys had an average egg count of 4234 EPG (light intensity). Out of the 7 infected girls, 6 had light intensity and 1 had moderate intensity. All the girls had an average of 4838 EPG (light intensity). Although both boys and girls had light intensity, the latter had a slightly higher average egg count. Nonetheless, the association between intensity of intestinal helminths and gender was not statistically significant (*p* = 0.906).

### 3.4. Risk Factors for Intensity of *Ascaris lumbricoides*

For factors affecting intensity of intestinal helminths, a statistically significant association was observed between intensity of intestinal helminths and factors like school location, knowledge of washing hands before eating, and awareness of washing hands after visiting the toilet as determined by Chi-square.

There was no statistically significant association between intensity of intestinal helminths for residence, method of faecal disposal, source of water, method of treating water for drinking, deworming interval, pupils' age, and pupils' gender as determined by Chi-square (Table 5).

## 4. Discussion

### 4.1. Prevalence of *Ascaris lumbricoides* in Lugari Subcounty

The overall prevalence of intestinal helminths observed in the current study population was very similar to that of Odiere and Odhiambo [16] in Kisumu, Western Kenya. The findings of the prevalence of intestinal helminths of the study are consistent with two similar studies carried out in Bondo district, western part of Kenya [17] and Sudan [25] in Southern Sudan that showed a prevalence of 12.9% and 12.3%, respectively. This was linked to geographical variations and socioeconomical and hygienic conditions of the population under consideration. The present study results are almost like a recent study carried out in three subcounties: Kakamega-Central, Kakamega-South, and Kakamega-East by Ngojo et al. [18]. In their study, *A*. *lumbricoides* was the most prevalent with a higher prevalence of 43.5% [18].

The low prevalence in the current study might be a result of school intestinal helminth prevention programmes by deworming. It is also likely that the environment is less contaminated, and the preschool pupils had knowledge on hand washing after using the toilet especially in the urban setting. Most urban schools have water with detergent provided for use outside the latrines. The absence of hookworms could be because the third larval stage has a lower life expectancy (3-10 days) unlike *A. lumbricoides* eggs that have several months of infective period [15]. There was a prevalence of 4.9% for *A. lumbricoides* and 7.7% for *T. trichiura* [16], and in a number of studies, *A. lumbricoides* infections were more prevalent than other intestinal helminths like *T. trichiura* and hookworms [26]. Moreover, *T. trichiura* eggs last for a shorter time in the soil than those of *A. lumbricoides* because they are very sensitive to desiccation because they do not have a thick sticky albumin coating that would make them adhere to different surfaces. They are easily swept away or washed to lower layers due to their smooth surface coating [27].

Low prevalences of *A. lumbricoides* of 4.6%, 16.2%, 12.4%, and 8.7% [3, 16, 26], respectively, have been reported from Western Kenya in the recent past compared to earlier studies where the prevalence was as high as 42.5% [28]. This is evident that the school deworming programme which was effected in Kenya in 1998 is effective [29, 30]. Multiple infections of intestinal helminths were not observed in this study which is a significant observation, and it could be credited to the impact of the school-based adopted control programme. Also, low prevalence of *A. lumbricoides* could be due to community access to improved hygiene practices as well as improved health service delivery. Generally, hookworms have a slower rate of infections compared to *A. lumbricoides.*

Studies on intestinal helminth infection prevalence from endemic countries indicate that ascariasis is more prevalent as compared to other intestinal helminths [31]. In this study, the prevalence of ascariasis was slightly more in boys than girls, but it was not statistically significant contrary to a study in Nigeria that indicated that it was [31]. This can be attributed to the fact that boys do participate in more outdoor activities like playing football with bare feet. This makes boys more infected than their female counterparts. However, another study [32] revealed an observation contrary to such findings where intestinal helminth infections were more common in females than in males.

Preschool children living near the river presented with higher prevalence of *A. lumbricoides* infections compared to children living in residential areas away from the river. Although there is no study that links intestinal helminth infections with closeness to the river, lowlands and upland tend to show differences in intestinal helminth infections [27]. This can be due to rain water carrying infected soil from other areas to the lowlands. The use of water from the river may increase infections further as observed in Colombia, with a trend in prevalence ranging between 0.6% and 2.4% for *A. lumbricoides* [33].

### 4.2. Risk Factors for the Prevalence of *Ascaris lumbricoides* in Lugari Subcounty

The Chi-square test revealed that the relationship between the prevalence of intestinal helminths and school location was statistically significant. To be precise, all the positive cases were from rural schools. The finding is not strange because poor hygiene and lack of piped water and other sanitary facilities in the rural area are strong determinants of high prevalence of intestinal helminths in rural schools [34]. The association between the area of residence and intestinal helminth infections was not statistically significant. In another study, intestinal helminths were found common in rural areas due to poor living conditions, lack of information about helminths, and poor sanitation [35, 36]. However, in the present study, a good number of individuals in rural areas practice hygienic standards.

Pupils who were not aware that they were supposed to wash hands before eating were 5 times more likely to be infected with intestinal helminths than pupils who knew. There was a statistically significant association between intestinal helminth infections and knowledge about washing hands before eating. Over 60% of the infected children did not know that they were supposed to wash hands before eating. However, over 30% knew that they were supposed to wash hands before eating. Although the questionnaire did not test the knowledge of using soap to wash hands before eating, the difference may be due to the fact that those who knew that they were supposed to wash hands did not use detergents or soap. It is also likely that they used untreated or contaminated water to wash their hands leading to intestinal helminth infections because persons who washed hands without soap had 2.6 times likelihood of being infected with *A. lumbricoides* compared with individuals who used soap.

The pupils who did not have any type of toilet at home were 4 times more likely to be infected with intestinal helminths than pupils who had toilets at home. The association between the prevalence of intestinal helminths and techniques of faecal disposal was not statistically significant. All the positive cases either relieved themselves in pit latrines and the bush. This study is in consonance with previous studies which showed that child latrine use is associated with intestinal helminth infections [21, 37]. Pupils who used pit latrines had 68.8% while those who used flush toilets had 12.5% of the positive cases. Interestingly, pupils who did not have any toilet or relieved themselves in the bush had 18.8% positive cases. That implied that despite the lack of latrine being one of the risk factors that expose children to *A. lumbricoides*, hygienic practices and other factors contribute to a high prevalence among pupils who use pit latrines [38].

Pupils who were not aware that they were supposed to wash hands after visiting the toilet were 4 times more likely to be infected with intestinal helminths than pupils who were aware. The association between the prevalence of intestinal helminths and awareness that one is supposed to wash hands after visiting the toilet was statistically significant. More of the infected children did not know that they were supposed to wash hands after visiting the toilet. This implies that improper hygienic practices like failure to wash hands after visiting the toilet could be the probable cause of autoinfection. Proper washing of hands after visiting the toilet can lower the risk of intestinal helminth infection by minimizing the shedding of helminth eggs into the surrounding environment, leading to reduced transmission [39].

Pupils who used water from both covered and uncovered wells were less likely to be infected than those who used water from the river, springs, and pipes. The number of pupils with intestinal helminths is 3-fold greater if a pupil uses water that is not treated than that who uses treated water. There was no statistically significant relationship between the prevalence of intestinal helminth infections and sources of water. This does not agree with other studies which indicated that intestinal helminth infections are high in children who drink unsafe water as compared to those who drink safe water [40]. More than a half of the pupils with intestinal helminth infections came from families in which people use river as a source of water which is a well-documented risk factor for intestinal helminth infections [5]. When water from wells and rivers comes in contact with overflowing drains and waste water or with open defecation, optimum conditions for the growth and development of the ova of *A. lumbricoides* are provided which lead to spreading of intestinal helminths especially *A. lumbricoides* [41].

The number of pupils with intestinal helminths was 3 times greater if a pupil used untreated water than the one who used treated water. This study confirms that the relationship between the prevalence of intestinal helminths and methods of treating water for drinking is not significant. This contradicts a study in Uganda which showed that failure to treat water before use can contribute to increased intestinal helminth infections [42]. However, treatment of domestic water has a positive impact on the prevalence of intestinal helminths [5]. This finding can be attributed to the fact that infections cut across board among children whose parents/guardians treat water and those who do not treat.

Pupils who were dewormed at intervals of 3 to 6 months had equal chances of being infected with intestinal helminths. The number of intestinal helminth infections among pupils who were dewormed annually and those who were not dewormed is 5-fold and 4-fold (respectively) greater than that of pupils who were dewormed at intervals of between 3 and 6 months. The current study confirms that the association between the prevalence of intestinal helminths and intervals of deworming is not statistically significant. Deworming on a regular basis has been effective in lowering intestinal helminth infections in a cost-efficient way because antihelminthic drugs can reduce the transmission of intestinal helminth infections by reducing worm load and production of intestinal helminth eggs [43, 44].

Pupils who were 5 years old were 50% more likely to be infected with intestinal helminths than those who were 7 years old. Pupils who were 6 years old were 60% and twice more likely to be infected with intestinal helminths than their counterparts who were 5 and 7 years old, respectively. Although the association between age and prevalence of intestinal helminth infections was not statistically significant, at 5, 6, and 7 years old, there were 37.5%, 50%, and 12.5% of the positive cases, respectively. Above 8 years of age, no pupil was infected with intestinal helminths. Younger children are not grown enough to participate in more outdoor activities. Studies show that as children grow, there is an increased trend in the involvement of more outdoor activities. This can lead to increased prevalence up to around a certain age at which the children gain knowledge about hygienic practices. Some may become less active, hence a decrease in the prevalence of intestinal helminths [45]. The number of boys with intestinal helminths was 20% greater than that of girls. Despite the fact that the relationship between the prevalence of intestinal helminths and gender was not statistically significant, boys had a slightly higher prevalence than girls. This finding does not agree with a past investigation which indicated that girls are more vulnerable to intestinal helminthiasis than boys [32].

### 4.3. Intensity of *Ascaris lumbricoides* in Lugari Subcounty

Most of the infected preschool children had a light to moderate infection intensity of *A. lumbricoides* infections. This difference may be due to increased reinfection in girls because 11.1% of the parents/guardians to girls had not heard about intestinal helminthiasis compared to 3% of parents/guardians to boys. Another reason could be the fact that 12.7% of the girls did not know that hands should be washed before eating compared to only 7.5% of their male counterparts. Additionally, over a third of the girls were not aware that they were required to wash hands after visiting the toilet compared to only a quarter of boys. Also, 12.7% of the parents/guardians to girls were not aware of deworming programmes compared to 6% of parents/guardians to boys. The last reason could be because 25.4% of the girls were not dewormed compared to 16.4% of the boys. Other studies have also concluded that girls have a higher average intensity, compared to their boy counterparts [46].

Although both boys and girls had light intensity, the latter had a slightly higher average egg count. Although the intensity was generally low, 24.5% of the participants only sieved water before drinking and 14.7% of them indicated that they did not treat or sieve water, but they instead use it directly. If no action is taken, this can increase the rate of infection which might lead to higher intensities of intestinal helminths in the future.

### 4.4. Risk Factors for the Intensity of *Ascaris lumbricoides* in Lugari Subcounty

The association between intensity of intestinal helminths and method of faecal disposal was not statistically significant. This is not a surprise because about 90% of the positive cases were recorded among children who use pit latrines and those who do it in the bush. Inadequate latrine access and improper use of the same can contribute to a high intensity of intestinal helminths [47, 48].

The association between intensity of intestinal helminths and deworming intervals was not statistically significant. People who dewormed their children once a year and those who did not deworm constituted 75% of the total infections. Regular deworming is known to reduce the transmissibility of intestinal helminths by reducing worm load and production of eggs [44].

The association between intensity of intestinal helminths and school location was statistically significant. All the positive cases were from rural schools. Lack of clean water and proper health amenities in rural areas are good indications of a high intensity of intestinal helminths [34]. These results are similar to a past study which concludes that residence and intensity of intestinal helminths are related due to features of the environment like unclean water [49].

The intensity of intestinal helminth infections and methods of treating water for drinking are not statistically significant, 70% of the positive cases comprised of those who failed to treat. Untreated water increases the intensity of intestinal helminths [5].

The association between intensity of intestinal helminths and awareness of washing hands before eating was statistically significant. Not all the preschool pupils who were aware that they were required to wash hands actually implemented the knowledge. Also, it seems that those who practiced hand washing did not do it properly. Research shows that correct washing of hands with appropriate detergents, after defecation or before meals, can lead to a decreased intensity of intestinal helminth infections [45].

The relationship between intensity of intestinal helminths was statistically significant. Despite the fact that both boys and girls had an average of light intensity, the latter had a slightly higher egg count than the former. This is consistent with another study which concluded that girls have a higher average intensity compared to their boy counterparts [50].

The association between intensity of intestinal helminths and awareness of washing hands before eating was statistically significant. This agrees with a study carried out in Western Uganda [50] which concluded that awareness of washing hands after visiting the toilet is one of the factors associated with intensity of intestinal helminths.

This study revealed a statistically insignificant association between intensity of intestinal helminths and residence. This agrees with the study done in Thika, Central Kenya [18], where intensity of intestinal helminth infection was significantly associated with area of residence. In the study, intensity was significantly high in rural residence. Although the differences were not statistically significant in the current study, light to moderate intensity was recorded from pupils in rural residence while no single infection was recorded from pupils who stayed in urban areas.

Intensity of intestinal helminths and sources of water were not statistically significantly associated. This observation contradicts another study [28] that concluded that intensity of intestinal helminth infections was significantly different in individuals using different sources of water.

The association between intensity of intestinal helminths and pupils' age is statistically insignificant. This contradicts a study in Tanzania [51] which indicated that intensity of intestinal helminths was significantly different among age groups.

## 5. Conclusion

Although a low prevalence light intensity was observed and only one species, *Ascaris lumbricoides*, was identified in this study, the prevalence of *A*. *lumbricoides* among preschool pupils was significantly associated with the method of faecal disposal, school location, awareness that one is supposed to wash hands before eating, source of water, and deworming interval. We recommend that stool collection to be done during different seasons. Although the Kato-Katz method is a fast method, researchers should consider other techniques because Kato-Katz is less sensitive [52, 53]. Thus, more efforts should be made to educate the preschool pupils and parents/guardians to avoid the build-up of the parasitic load in the environment to achieve zero helminthiases in the country.

## Figures and Tables

**Table 1:** Prevalence of *Ascaris lumbricoides* in preschool pupils.

Primary school	Total participants			Prevalence of *Ascaris lumbricoides*		
	Boys + girls	Boys	Girls	Boys + girls	Boys	Girls
Rural schools	64.6% (84/130)	47.7% (40/84)	52.3% (44/84)	19% (16/84)	22.5% (9/40)	16% (7/44)
Majengo	9.2% (12/130)	33.3% (4/12)	66.7% (8/12)	8.3% (1/12)	25% (1/4)	0
Sirende	27.7% (36/130)	52.8% (19/36)	47.2% (17/36)	30.6% (11/36)	26.3% (5/19)	35.3% (6/17)
Mapengo	9.2% (12/130)	41.7% (5/12)	58.3% (7/12)	0	0	0
Chetambe	18.5% (24/130)	50% (12/24)	50% (12/24)	16.67% (4/24)	25% (3/12)	8.3% (1/12)
Periurban schools	35.4% (46/130)	58.7% (27/46)	41.3% (19/46)	0	0	0
Lumakanda	11.5% (15/130)	80% (12/15)	20% (3/15)	0	0	0
Lugari	11.5% (15/130)	53.3% (8/15)	46.7% (7/15)	0	0	0
Musembe	7.7% (10/130)	40% (4/10)	60% (6/10)	0	0	0
Maturu	4.6% (6/130)	50% (3/6)	50% (3/6)	0	0	0

**Table 2:** Association between different factors and prevalence of *A. lumbricoides.*

Variable	*A. lumbricoides* infection			Prevalence ratio	
	Yes	No	Total	Ratio of infection	Ratio compared to the baseline group
Knowledge of washing hands before eating					
No	6	7	13	6/13 = 0.462	0.462/0.085 = 5.4
Yes	10	107	117	10/117 = 0.085	0.085/0.085 = 1
Toilet at home					
No	3	4	7	3/7 = 0.429	0.429/0.106 = 4.1
Yes	13	110	123	13/123 = 0.106	0.106/0.106 = 1
Knowledge of washing hands after visiting the toilet					
No	10	27	37	10/37 = 0.27	0.270/0.065 = 4.2
Yes	6	87	93	6/93 = 0.065	0.065/0.065 = 1
Source of water					
River	8	26	34	8/34 = 0.235	0.235/0.056 = 4.2
Many	1	6	7	1/7 = 0.143	0.143/0.056 = 2.6
Spring	1	10	11	1/11 = 0.091	0.091/0.056 = 1.6
Piped	1	8	9	1/9 = 0.111	0.111/0.056 = 2
Covered well	4	46	50	4/50 = 0.08	0.080/0.056 = 1.4
Uncovered well	1	17	18	1/18 = 0.056	0.056/0.056 = 1
Treatment of water for drinking					
No	11	45	56	11/56 = 0.196	0.196/0.065 = 3
Yes	5	72	77	5/77 = 0.065	0.065/0.065 = 1
Deworming interval					
12 months	6	19	25	6/25 = 0.24	0.240/0.053 = 4.6
Do not deworm	6	21	27	6/27 = 0.222	0.222/0.053 = 4.2
6 months	1	18	19	1/19 = 0.053	0.053/0.053 = 1
3 months	3	52	55	3/55 = 0.055	0.055/0.053 = 1
Pupils' age					
5	6	40	46	6/46 = 0.13	0.130/0.087 = 1.5
6	8	35	43	8/43 = 0.186	0.186/0.087 = 2.1
7	2	21	23	2/23 = 0.087	0.087/0.087 = 1
Pupils' gender					
Male	9	58	67	9/67 = 0.134	0.134/0.111 = 1.2
Female	7	56	63	7/63 = 0.111	0.111/0.111 = 1

**Table 3:** Association between prevalence and risk factors.

Variable	+ve % (*n*/16)	-ve % (*n*/114)	Chi *p* value
School location			
Periurban	0	40.4% (46)	0.002^∗^
Rural	100% (16)	59.6% (68)	
Place of residence			
Rural	100% (16)	94.7% (108)	0.347
Urban	0	5.3% (6)	
Knowledge of washing hands before eating			
Yes	62.5% (10)	107 (93.9%)	0.001^∗^
No	37.5% (6)	6.1% (7)	
Faecal disposal			
Pit latrine	81.3% (13)	86% (98)	
Flush toilet	0	9.6% (11)	0.051
Flush & pit	0	0.9% (1)	
Bush	18.7% (3)	3.5% (4)	
Knowledge of washing hands after visiting the toilet			
Yes	37.5% (6)	76.3% (87)	0.001^∗^
No	62.5% (10)	23.7% (27)	
Source of water			
River	8 (50%)	22.8% (26)	
Piped water	6.3% (1)	7% (8)	
Uncovered well	6.3% (1)	14.9% (17)	0.336
Covered well	25% (4)	40.4% (46)	
Many	6.3% (1)	5.3% (6)	
Spring water	6.3% (1)	8.8% (10)	
Method of treating water for drinking			
Sieving	43.8% (7)	29.8% (34)	
Chemical	18.8% (3)	46.5% (53)	
Boiling	12.5% (2)	14% (16)	0.175
Many	0	2.6% (3)	
Do not treat	25% (4)	9.6% (11)	
Deworming interval			
3 months	18.8% (3)	45.6% (52)	
6 months	6.3% (1)	15.8% (18)	
12 months	37.5% (6)	16.7% (19)	0.051
Do not deworm	37.5% (6)	18.4% (21)	
Uncertain	0	3.5% (4)	
Pupils' age			
5	37.5% (6)	35.1% (40)	
6	50% (8)	30.7% (35)	
7	12.5% (2)	18.4% (21)	0.354
8	0	9.6% (11)	
9	0	6.1% (7)	
Pupils' gender			
Male	56.25% (9)	50.9% (58)	0.687
Female	43.75% (7)	49.1% (56)	

^∗^Significant variables at 0.05.

**Table 4:** Intensity of *Ascaris lumbricoides* Lugari Subcounty.

Classification strata and categories	Total cases in boys			Total cases in girls			Total cases in boys + girls		
	Light	Moderate	Heavy	Light	Moderate	Heavy	Light	Moderate	Heavy
(1) Schools									
Majengo	1	0	0	0	0	0	0	0	0
Sirende	4	1	0	5	1	0	9	2	0
Chetambe	3	0	0	1	0	0	4	0	0
(2) Wards									
Lumakanda	1	0	0	0	0	0	1	0	0
Lugari	4	1	0	5	1	0	9	2	0
Luandeti	3	0	0	1	0	0	4	0	0
(3) Overall	8	1	0	6	1	0	14	2	0

**Table 5:** Association between intensity and risk factors.

Variable	Infected	Not infected	*p* value
School location			
Periurban	0	46/114 (40.4%)	0.007^∗^
Rural	16/16 (100%)	68/114 (59.6%)	
Place of residence			
Rural	16/16 (100%)	108/114 (94.7%)	0.643
Urban	0	6/114 (5.3%)	
Knowledge of washing hands before eating			
Yes	10/16 (62.5%)	107/114 (93.9%)	0.001^∗^
No	6/16 (37.5%)	7/114 (6.1%)	
Faecal disposal			
Pit latrine	13/16 (81.3%)	98/114 (86%)	
Flush toilet	0	11/114 (9.6%)	0.154
Flush & pit	0	1/114 (0.9%)	
Bush	3/16 (18.7%)	4/114 (3.5%)	
Knowledge of washing hands after visiting the toilet			
Yes	6/16 (37.5%)	87/114 (76.3%)	0.003^∗^
No	10/16 (62.5%)	27/114 (23.7%)	
Source of water			
River	8/16 (50%)	26/114 (22.8%)	
Piped water	1/16 (6.3%)	8/114 (7%)	
Uncovered well	1/16 (6.3%)	17/114 (14.9%)	0.299
Covered well	4/16 (25%)	46/114 (40.4%)	
Many	1/16 (6.3%)	6/114 (5.3%)	
Spring water	1/16 (6.3%)	10/114 (8.8%)	
Method of treating water for drinking			
Sieving	7/16 (43.8%)	34/114 (29.8%)	
Chemical	3/16 (18.8%)	53/114 (46.5%)	
Boiling	2/16 (12.5%)	16/114 (14%)	0.427
Many	0	3/114 (2.6%)	
Do not treat	4/16 (25%)	11/114 (9.6%)	
Deworming interval			
3 months	3/16 (18.8%)	52/114 (45.6%)	
6 months	1/16 (6.3%)	18/114 (15.8%)	
12 months	6/16 (37.5%)	19/114 (16.7%)	0.161
Do not deworm	6/16 (37.5%)	21/114 (18.4%)	
Uncertain	0	4/114 (3.5%)	
Pupils' age			
5	6/16 (37.5%)	40/114 (35.1%)	
6	8/16 (50%)	35/114 (30.7%)	
7	2/16 (12.5%)	21/114 (18.4%)	0.522
8	0	11/114 (9.6%)	
9	0	7/114 (6.1%)	
Pupils' gender			
Male	9/16 (56.25%)	58/114 (50.9%)	0.906
Female	7/16 (43.75%)	56/114 (49.1%)	

^∗^Significant variables at 0.05.

## Data Availability

Due to the sensitive nature of the questions asked in this study, survey respondents were assured raw data would remain confidential and would not be shared.
